# Prehospital stroke-scale machine-learning model predicts the need for surgical intervention

**DOI:** 10.1038/s41598-023-36004-8

**Published:** 2023-06-05

**Authors:** Yoichi Yoshida, Yosuke Hayashi, Tadanaga Shimada, Noriyuki Hattori, Keisuke Tomita, Rie E. Miura, Yasuo Yamao, Shino Tateishi, Yasuo Iwadate, Taka-aki Nakada

**Affiliations:** 1 Department of Neurosurgery, Chiba Municipal Kaihin Hospital, Chiba, Japan; 2 Department of Neurological Surgery, Graduate School of Medicine, Chiba University, Chiba, Japan; 3 Department of Emergency and Critical Care Medicine, Chiba University Graduate School of Medicine, 1-8-1 Inohana, Chuo, Chiba 260-8677 Japan; 4 SMART119 Inc., 7th Floor, Chiba Chuo Twin Building No. 2, 2-5-1 Chuo, Chiba, Japan

**Keywords:** Stroke, Stroke

## Abstract

While the development of prehospital diagnosis scales has been reported in various regions, we have also developed a scale to predict stroke type using machine learning. In the present study, we aimed to assess for the first time a scale that predicts the need for surgical intervention across stroke types, including subarachnoid haemorrhage and intracerebral haemorrhage. A multicentre retrospective study was conducted within a secondary medical care area. Twenty-three items, including vitals and neurological symptoms, were analysed in adult patients suspected of having a stroke by paramedics. The primary outcome was a binary classification model for predicting surgical intervention based on eXtreme Gradient Boosting (XGBoost). Of the 1143 patients enrolled, 765 (70%) were used as the training cohort, and 378 (30%) were used as the test cohort. The XGBoost model predicted stroke requiring surgical intervention with high accuracy in the test cohort, with an area under the receiver operating characteristic curve of 0.802 (sensitivity 0.748, specificity 0.853). We found that simple survey items, such as the level of consciousness, vital signs, sudden headache, and speech abnormalities were the most significant variables for accurate prediction. This algorithm can be useful for prehospital stroke management, which is crucial for better patient outcomes.

## Introduction

Early treatment of stroke with large vessel occlusion (LVO) requires rapid transport to a thrombectomy-capable hospital for early recanalization^1,2^. Similarly, timely transport to specialized facilities is critical for treating subarachnoid haemorrhage (SAH) and intracerebral haemorrhage (ICH), as it can significantly improve patient outcomes^3,4^. The American Stroke Association recommends recognizing stroke accurately, activating emergency medical services (EMS), triaging to the appropriate hospital, and designating a competent stroke centre^5^. However, accurately diagnosing stroke in prehospital settings can be challenging for EMS personnel due to resource constrains and similarity of symptoms between different types of strokes, potentially leading to delays in hospital arrival or misdiagnosis. To address this issue, various prehospital scales targeting LVO have been developed to aid in determining indications for thrombectomy^6–9^, and the prehospital diagnosis of SAH and ICH is also important for acute stroke treatment^10,11^. Moreover, recent advances in machine learning (ML) and deep learning (DL) models have shown promising results in improving prehospital diagnosis scales^12–14^.

In the medical field, ML and DL models have demonstrated their potential in computer-aided diagnosis, helping healthcare professionals make accurate and timely diagnoses. For instance, DL-based image classification has been used to diagnose diseases such as pneumonia, breast cancer and lung cancer^15–17^. The ML-driven segmentation of medical images has enabled the detection of regions of interest^18^, and feature extraction techniques have been used to extract relevant features from medical images or other data for developing diagnostic tools^19,20^. ML models, as decision support systems, have been developed and used to assist clinicians in diagnosing and treating diseases [e.g., heart diseases^21^]. To improve the accuracy of machine learning models, there are generally three methods of hyperparameter optimization. Grid Search, Random Search, and Bayesian Optimisation. Grid search is simple and easy to implement. However, it is computationally expensive when the hyperparameter space is large, and it doesn't learn adaptively from previous iterations. Random search allows to explore the hyperparameter space more efficiently compared to grid searching. But because it's random, there is no guarantee that the best hyperparameter combination will be found. Bayesian optimization efficiently explores the hyperparameter space by using a probabilistic model to guide the search. It adapts the search based on previous evaluations, improving efficiency. It uses a learning function to select the next hyperparameter configuration to evaluate, balancing exploration and exploitation.

Building upon these recent advances, our study aimed to develop an ML-driven decision support system to help EMS personnel diagnose patients with consistent accuracy. We collected stroke-related information from the records of patients with suspected stroke who were transported by EMS to a single secondary medical care area as part of the Smart119 project. In our previous paper, we analysed the data using ML models and presented a stroke prediction scale that includes the diagnosis of stroke categories^22^. In this work, considering that the prehospital selection of patients requiring surgical treatment, rather than the diagnosis of stroke subtypes, would contribute to more appropriate transport, we examined the prehospital predictive diagnosis of patients who actually required surgical intervention based on case data from the Smart119 project. Our findings suggest that by integrating ML into prehospital decision support for EMS personnel, it is possible to improve patient outcomes by enabling appropriate and timely transport of patients requiring stroke surgical treatment.

## Results

### Baseline characteristics and outcomes

Patient characteristics and clinical findings in the study model are shown in Tables 1, 2, 3, S1–S3. There was no significant difference in patient background between the two groups; however, the intervention group had a significantly shorter time from onset to command (Table 1). In terms of the level of consciousness, treatment intervention was less common in patients with code alerts (Japan Coma Scale [JCS] 0, Glasgow Coma Scale [GCS] E4V5M6) and significantly more common in patients with codes JCS 3–100 and GCS E3/V2/M4-5 (Table 2). Vital signs and symptoms that required considerably more intervention were sudden headache, vomiting, hemiparesis, conjugate deviation, aphasia, and dysarthria (Table 3).

**Table 1:** Baseline characteristics in the training cohort.

	Treatment (N = 131)	No treatment (N = 634)	*p* value
Age, years	74.0 (62.0–81.0)	74.0 (62.0–82.0)	0.477
Male sex, n(%)	71 (54.2%)	377 (59.5%)	0.310
Past medical history			
Intracranial haemorrhage, n(%)	3 (2.5%)	36 (6.1%)	0.179
Anticoagulant/Antiplatelet therapy, n(%)	7 (6.0%)	62 (11.1%)	0.139
ADL indipendent, n(%)	110 (87.3%)	515 (86.1%)	0.835
Atrial fibrillation, n(%)	6 (4.9%)	46 (7.7%)	0.360
Hypertension, n(%)	51 (41.5%)	284 (47.0%)	0.304
Diabetes mellitus, n(%)	15 (12.4%)	106 (17.6%)	0.205
Cerebral infarction, n(%)	20 (16.5%)	135 (22.5%)	0.181
Time course and onset timing			
Time from onset to emergency call	19.5 (7.0–105.0)	54.5 (13.0–250.0)	< 0.001
Onset timing Monday	20 (15.3%)	83 (13.1%)	0.601
Onset timing Tuesday	20 (15.3%)	71 (11.2%)	0.246
Onset timing Wednesday	15 (11.5%)	86 (13.6%)	0.611
Onset timing Thursday	17 (13.0%)	82 (12.9%)	1.000
Onset timing Friday	21 (16.0%)	82 (12.9%)	0.421
Onset timing Saturday	18 (13.7%)	110 (17.4%)	0.379
Onset timing Sunday	20(15.3%)	120 (18.9%)	0.389

Data are presented as median and interquartile range for continuous variables.

*P* values were calculated using Pearson’s chi-square test or the Mann–Whitney U test.

**Table 2:** Level of consciousness in the training cohort.

	Treatment (N = 131)	No treatment (N = 634)	*p* value
Japan coma scale			
JCS 0, n (%)	28 (21.4%)	314 (49.5%)	< 0.001
JCS I-1, n (%)	22 (16.8%)	82 (12.9%)	0.301
JCS I-2, n (%)	9 (6.9%)	44 (6.9%)	1.000
JCS I-3, n (%)	31 (23.7%)	81 (12.8%)	0.002
JCS II-10, n (%)	13 (9.9%)	29 (4.6%)	0.025
JCS II-20, n (%)	7 (5.3%)	11 (1.7%)	0.030
JCS II-30, n (%)	2 (1.5%)	6 (0.9%)	0.902
JCS III-100, n (%)	9 (6.9%)	13 (2.1%)	0.007
JCS III-200, n (%)	6 (4.6%)	31 (4.9%)	1.000
JCS III-300, n (%)	4 (3.1%)	23 (3.6%)	0.949
Glasgow coma scale			
GCS (E) = 4, n (%)	79 (60.3%)	497 (78.4%)	< 0.001
GCS (E) = 3, n (%)	29 (22.1%)	66 (10.4%)	< 0.001
GCS (E) = 2, n (%)	8 (6.1%)	16 (2.5%)	0.062
GCS (E) = 1, n (%)	15 (11.5%)	55 (8.7%)	0.403
GCS (V) = 5, n (%)	37 (28.2%)	336 (53.0%)	< 0.001
GCS (V) = 4, n (%)	30 (22.9%)	122 (19.2%)	0.404
GCS (V) = 3, n (%)	11 (8.4%)	28 (4.4%)	0.095
GCS (V) = 2, n (%)	21 (16.0%)	37 (5.8%)	< 0.001
GCS (V) = 1, n (%)	30 (22.9%)	107 (16.9%)	0.131
GCS (M) = 6, n (%)	82 (62.6%)	482 (76.0%)	0.002
GCS (M) = 5, n (%)	19 (14.5%)	54 (8.5%)	0.050
GCS (M) = 4, n (%)	16 (12.2%)	40 (6.3%)	0.029
GCS (M) = 3, n (%)	1 (0.8%)	7 (1.1%)	1.000
GCS (M) = 2, n (%)	3 (2.3%)	18 (2.8%)	0.955
GCS (M) = 1, n (%)	8 (6.1%)	29 (4.6%)	0.603

JCS (Japan coma scale), GCS (Glasgow coma scale).

Data are presented as median and interquartile range for continuous variables.

*P* values were calculated using Pearson’s chi-square test or the Mann–Whitney U test.

**Table 3:** Vital signs and symptoms in the training cohort.

	Treatment (N = 131)	No treatment (N = 634)	*p* value
Vital signs			
Heart rate	78.0 (66.0–92.5)	84.0 (72.0–100.0)	0.001
Arrhythmia	33 (28.9%)	131 (23.8%)	0.295
Systolic blood pressure	173.0 (154.0–202.2)	174.0 (151.0–197.2)	0.570
Diastolic blood pressure	97.0 (84.8–112.8)	97.0 (83.0–110.2)	0.446
Body temperature	36.4 (36.0–36.8)	36.5 (36.2–36.8)	0.023
Oxygen saturation	97.0 (96.0–98.0)	98.0 (96.0–99.0)	0.226
Symptoms			
Vomiting, n (%)	29 (22.1%)	70 (11.1%)	0.001
Dizziness, n (%)	11 (12.5%)	63 (12.2%)	1.000
Numbness, n (%)	9 (10.2%)	95 (19.6%)	0.051
Convulsion, n (%)	1 (0.8%)	37 (6.0%)	0.030
Upper limb paralysis, n (%)	79 (67.5%)	309 (54.7%)	0.014
Hemiparalysis, n (%)	59 (49.6%)	219 (38.2%)	0.028
Conjugate deviation, n (%)	32 (28.6%)	75 (13.5%)	< 0.001
Facial palsy, n (%)	33 (42.9%)	138 (31.7%)	0.075
Aphasia, n (%)	34 (37.0%)	96 (19.6%)	< 0.001
Dysarthria, n (%)	64 (77.1%)	212 (49.0%)	< 0.001
Unilateral spatial neglect, n (%)	2 (5.0%)	9 (3.3%)	0.928
Sudden headache or unconsciousness, n (%)	74 (56.5%)	226 (35.6%)	< 0.001
Sudden headache, n (%)	25 (23.1%)	33 (8.1%)	< 0.001

Data are presented as median and interquartile range for continuous variables.

*P* values were calculated using Pearson’s chi-square test or the Mann–Whitney U test.

### Prediction of prehospital stroke surgical intervention

**Figure 1:**
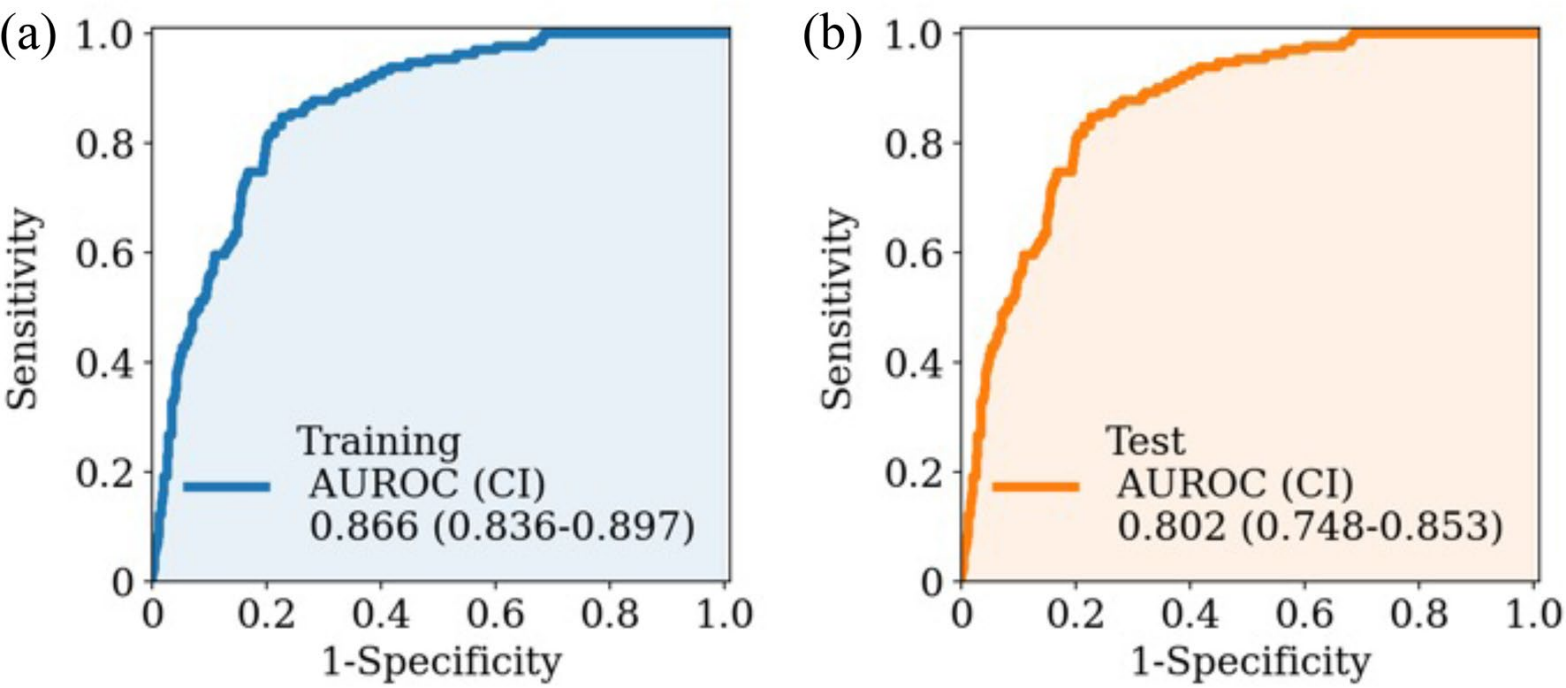
Area under the receiver operating characteristic curves of machine learning models. The receiver operating characteristic curve of prehospital prediction algorithms for stroke requiring surgical intervention is depicted with 1-specificity on the x-axis and sensitivity on the y-axis using the training cohort **(a)** and the test cohort **(b)**. The 95% confidence interval of the AUROC is also shown. *AUROC* area under the receiver operating characteristic curve.

**Figure 2:**
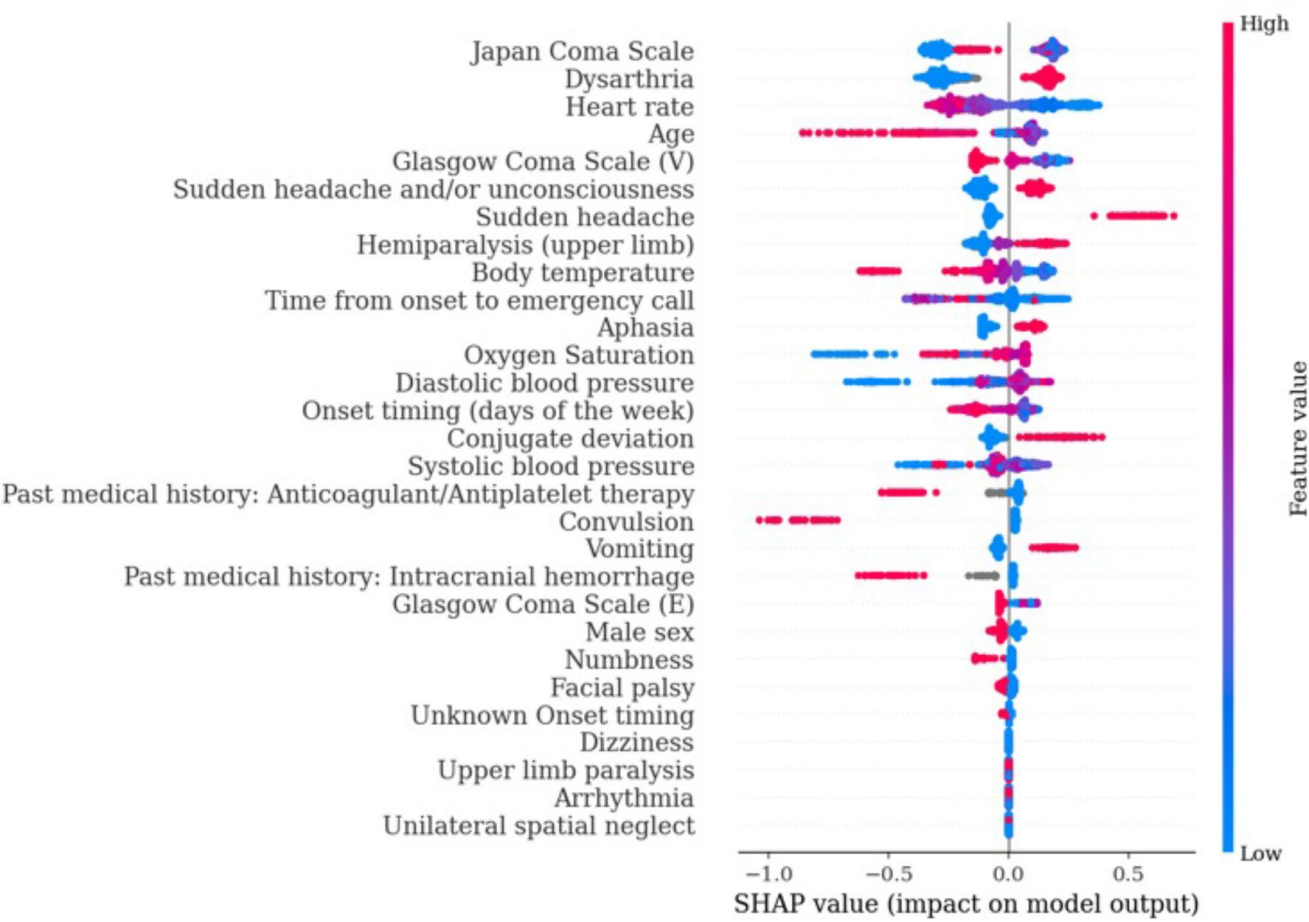
SHAP value of stroke surgical intervention. The impact of the features on the model output was expressed as the SHAP value. The features are placed in descending order according to their importance. The association between the feature value and SHAP value indicates a positive or negative impact of the predictors. The extent of the value is depicted as red (high) or blue (low) plots. *SHAP* SHapley Additive exPlanation.

Four popular ML algorithms were used to predict the need for stroke surgical intervention: eXtreme Gradient Boosting (XGBoost), Logistic Regression, Random Forest, and Support Vector Machine (SVM) as a representative of a gradient boosting algorithm, linear algorithm, tree algorithm, and dimensionality reducer and classifier (Table S4). In the training cohort, analysis using Random Forest predicted surgical intervention in stroke patients with high performance (an area under the receiver operating characteristic curve [AUROC] of 0.882, a sensitivity of 0.862, and a specificity of 0.746). When applied to the test cohort, the XGBoost model performed the best and predicted surgical intervention with higher scores than other models, achieving an AUROC of 0.802 (sensitivity 0.719, specificity 0.774) (Table 4, Fig. 1). The Shapley Additive exPlanation (SHAP) summary plot revealed that the major predictive contributors for stroke intervention were “Japan Coma Scale”, “dysarthria”, “heart rate”, “age”, “sudden headache and/or unconsciousness”, “Glasgow coma scale (V)”, “time from onset to emergency call”, “body temperature”, “aphasia”, and “oxygen saturation” (Fig. 2).

**Table 4:** Prehospital stroke prediction for intervention using XGBoost.

AUROC	Accuracy	Sensitivity	Specificity	F1-score
Training cohort				
0.866 (0.836–0.897)	0.786 (0.757–0.813)	0.840 (0.776–0.901)	0.774 (0.741–0.805)	0.573 (0.512–0.632)
Test cohort				
0.802 (0.748–0.853)	0.765 (0.72–0.804)	0.719 (0.606–0.826)	0.774 (0.728–0.820)	0.508 (0.418–0.590)

*AUROC* area under the receiver operating characteristic curve.

## Discussion

The present study demonstrated that a prehospital scale could predict stroke requiring surgical intervention with high accuracy. Although prehospital stroke diagnostic scales have been published in many countries and scales have been developed to determine the severity of stroke^23,24^, to the best of our knowledge, this is the first scale that predicts the need for surgical intervention. When surgical intervention is needed for any type of stroke, rapid transport is necessary, and hospitals need to be prepared for this. Therefore, this scale, which can predict the need for surgical intervention before hospital arrival, is very useful for rapid patient transport.

**Figure 3:**
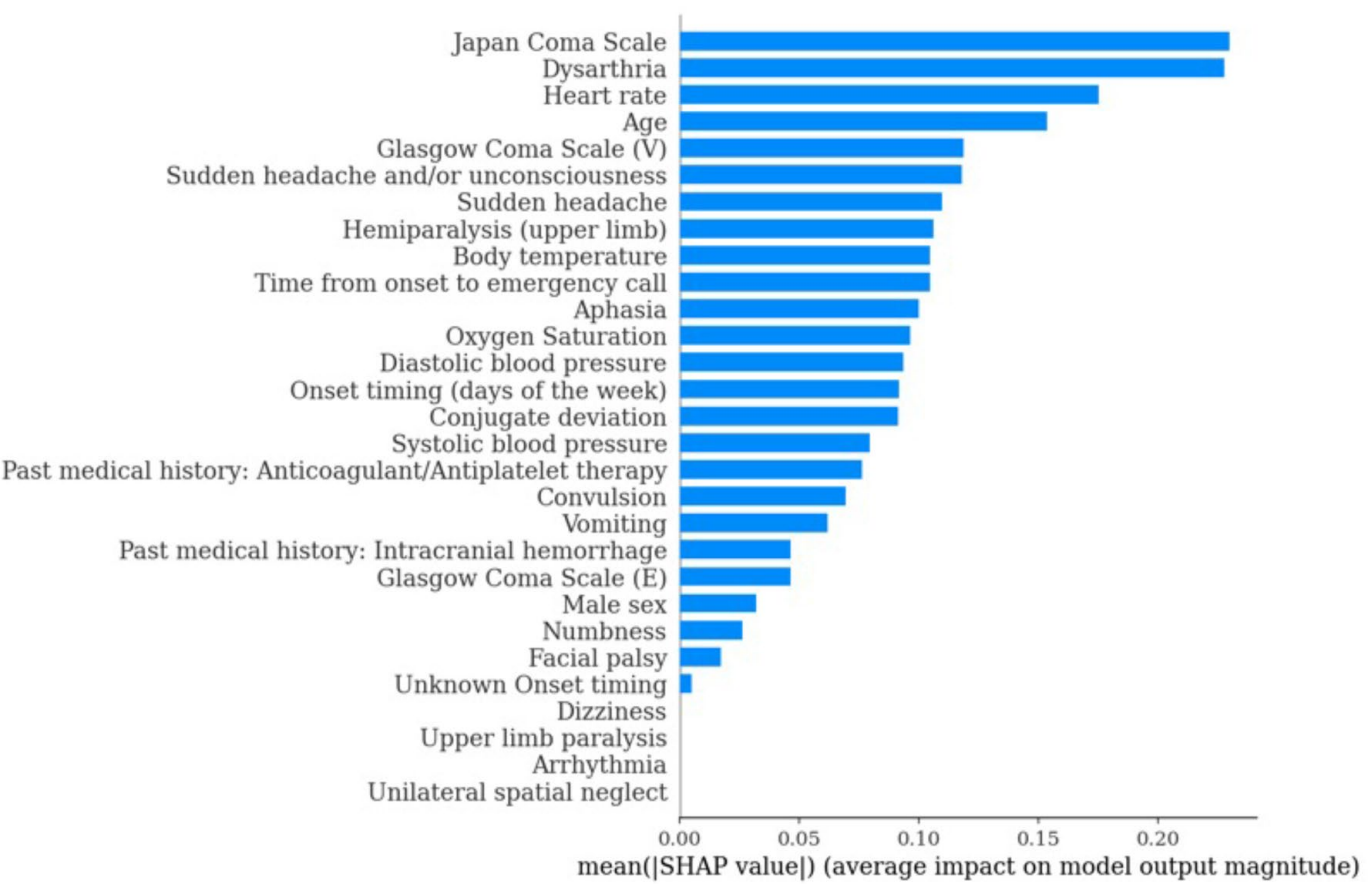
Feature importance. The impact of the features on the model output is expressed as the average of the absolute SHAP value. The larger the value, the more important is the feature for predicting stroke surgical intervention. *SHAP* SHapley Additive exPlanation.

The most important variables for diagnosis were found to be JCS, vitals (pulse, temperature, oxygen saturation), age, time from onset to emergency call, headache, and speech abnormalities. Interestingly, a detailed neurological examination was not among them (Fig. 3). These variables identified as most important variables are simple survey items, and we believe that the scale is composed of easily obtainable data that EMS teams routinely observe. Regarding the absence of a detailed neurological examination including paresis, it is interesting to note that the focus should be on other items that reflect disease severity since paresis and neurological deficits are observed even in minor strokes that do not require surgical intervention.

The prehospital stroke scales that have been published to date have enabled the diagnosis of LVO with high accuracy and stroke subtypes. However, in all of the scales, stroke-specific survey items were important, such as conjugate deviation and hemispatial neglect^25–27^. As noted above, these items were not included in the key variables in this scale, suggesting that its usefulness could be maintained even when these items were missing, such as in cases in which stroke was not suspected.

The novel prehospital scale developed in this study can predict the need for surgical intervention across all stroke diseases. In comparison, the Shonan Prehospital Scale (SPSS) is a score used at the municipal level to predict surgical intervention. The SPSS evaluates severe headache, impaired consciousness (JCS ≥ 10), and local symptoms (hemiplegia, facial paralysis, or abnormal speech), scoring 1 point if the onset is severe and 2 points if it is sudden onset. Comparative validation of the two models using the present data revealed that the newly developed model was superior to the other with an AUROC of 0.652 (sensitivity 0.880, specificity 0.425) (Table S5).

The patient information system utilized in the Smart119 project stores patient data gathered by EMS personnel via tablets. The interface of the system is equipped with an application that enables prehospital stroke diagnosis. We believe that the inclusion of this program into the existing system would reduce the time required for hospital selection and contribute to prompt and appropriate emergency transport.

This study has some limitations. First, the decision to initiate therapeutic intervention was made by the neurosurgeons at each participating hospital, which may have introduced variability across institutions. Second, although this was a multicentre study, the study was limited to a single metropolitan area in Japan. Hence, it is crucial to validate the algorithm’s high predictive value in other regions with distinct characteristics to increase its applicability across Japan. Fortunately, the medical region where this study was conducted (Chiba Prefecture) comprises diverse types of medical organizations, including urban type with multiple hospitals, independent type with one hospital as the main hospital, and depopulated type with no central hospital. The algorithm will be expanded to Chiba Prefecture as a whole and will be demonstrated in the future.

In conclusion, our algorithm serves as a prehospital stroke scale that can be easily completed by EMS personnel to predict the need for surgical intervention in patients with stroke. We firmly believe that our machine-learning-based scale holds significant value as predicting stroke intervention is important in determining a suitable transport destination considering their medical care system.

## Methods

### Study design and patient population

From September 2019 to January 2022, we conducted a study of patients who were transported by EMS for suspected stroke. The destination hospitals included all 12 medical institutions within the secondary care area that were equipped to transport stroke patients. We developed a surgical intervention prediction scale by retrospectively examining 1143 patients whose diagnosis and treatment plan could be ascertained at the transport site.

Surgical intervention was defined as aneurysmal neck clipping or coil embolization for SAH, haematoma removal, haematoma or ventricular drainage for ICH, administration of intravenous tissue plasminogen activator (tPA), mechanical thrombectomy, or other endovascular treatment for acute ischaemic stroke. The decision to perform interventions was made at the discretion of the neurosurgeon at each institution.

The Chiba University Hospital Certified Clinical Research Review Board approved this study (No. 2733) and waived the need for written informed consent in conformity with the Ethical Guidelines for Medical and Health Research Involving Human Subjects in Japan. We posted information about this study in each ambulance. We promptly excluded the collected data when a patient or family indicated that they did not wish to participate in this study.

### Selected variables

The survey items for analysis included patients’ characteristics, vital signs, symptoms, level of consciousness, and the 7 key parameters proposed by the Japanese Stroke Association. Details are as follows: (i) patients’ characteristics: age, sex, time from onset to emergency call, onset timing; (ii) vital signs: pulse, blood pressure (systolic/diastolic), body temperature, oxygen saturation; (iii) symptoms: vomiting, dizziness, cramps, numbness; (iv) level of consciousness: JCS, GCS (E, V, M); (v) previous medical history; (vi) important stroke parameters: conjugate deviation, hemispatial neglect by 4-finger method^25^, aphasia (call of glasses/clock), pulse irregularity, dysarthria, facial paralysis, upper and lower hemiparesis.

### Missing values

As our data had missing values, we performed imputations before building the ML models. First, we used domain knowledge to impute pairs of groups of features including (i) conjugate deviation and visual field defects (ii) dysarthria and facial paralysis; (iii) aphasia, GCS, JCS and other consciousness-related features; (iv) systolic and diastolic blood pressure values; and (v) paralysis-related features. For other numerical features, such as heart rate, body temperature, oxygen saturation, and time from onset to emergency call, we imputed with the median value of each feature. The rest of the features with missing values (all of them are categorical features) were left as they were since boosting models such as XGBoost support missing values and treat them as a separate category.

### Machine learning model development

We developed ML models using four different algorithms: XGBoost, Random Forest, Logistic Regression and SVM. To ensure a balanced distribution of surgical intervention categories, we randomly assigned 765 cases (70%) to a training cohort and 378 cases (30%) to a test cohort. The stroke types were classified into SAH, ICH, LVO, and other ischaemic stroke in both cohorts. The number of cases and the number of surgical interventions for each type are shown in Fig. S1.

The hyperparameters of the ML models were tuned by using an open-source hyperparameter optimization software framework called Optuna that employs Bayesian optimization algorithm techniques. Optuna helps us to find the best combination of parameters that maximize the model score by iterating the choice of parameters and evaluating the models obtained with those parameters. In each iteration, an evaluation of a model was performed with the scoring method AUROC through fivefold cross-validation.

### Statistical analysis

Model performance was measured in terms of the AUROC, sensitivity, specificity, and F1 score. Furthermore, the SHAP algorithm of the XGBoost model, which outperformed all other models, wa employed to interpret the contribution of each variable to the predictive model^28^. In this algorithm, the SHAP values are calculated by measuring the difference in model output resulting from the inclusion of a variable into the algorithm, providing insights into the impact of each variable on the output. In the SHAP plots, a violin plot was created for all data points associated with each feature, with higher values appearing red and lower values appearing blue. The violin plot is aligned with the SHAP value as the x-axis. Thus, the red/blue violin plot on the right (i.e., higher positive SHAP values) suggests that the higher/lower the value of that feature, the better the model predicts towards positive/negative effects.

Continuous values were expressed as medians (interquartile ranges), and categorical values were presented as absolute numbers and percentages. Two-sided P values less than 0.05 were considered indicative of statistical significance.

Analyses were performed using the open-source Python 3.7.15 package, XGBoost 1.5.1, Sciki-learn 1.0.2, Pandas 1.3.5, Optuna 2.10.1, and SHAP 0.41.0 package. (Python Licence: https://docs.python.org/3/license.html**,** XGBoost: https://github.com/dmlc/xgboost/blob/master/LICENSE, Scikit-learn: https://github.com/scikit-learn/scikit-learn/blob/main/COPYING**,** Pandas:http://pandas.pydata.org/pandasdocs/stable/getting_started/overview.html?highlight=license**,** Optuna: https://github.com/optuna/optuna/blob/master/LICENSE, SHAP: https://github.com/slundberg/shap/blob/master/LICENSE. All figures in this study were drawn using Matplotlib (3.2.2)^20,21^, a Python visualization package.

## Supplementary Information

Supplementary Information.

## Data Availability

The datasets used and analysed during our study are available from the corresponding author upon reasonable request.

## Abbreviations

LVO: Large vessel occlusion
EMS: Emergency medical services
SAH: Subarachnoid haemorrhage
ICH: Intracerebral haemorrhage
JCS: Japan coma scale
GCS: Glasgow coma scale
XGBoost: EXtreme gradient boosting
AUROC: Area under the receiver operating characteristic curve
tPA: Tissue plasminogen activator
SHAP: SHapley Additive exPlanation
