# Supplementary material for: Prehospital stroke-scale machine-learning model predicts the need for surgical intervention
Source: Sci Rep. 2023 Jun 5;13:9135. doi: 10.1038/s41598-023-36004-8 (PMC10241931; doi:10.1038/s41598-023-36004-8)
Supplement: Supplementary file 1 — Supplementary Information. [file 41598_2023_36004_MOESM1_ESM.pdf]

Supplemental materials

Supplementary Information

Supplementary Figure S1. Study flowchart

Supplementary Table S1. Baseline characteristics in the test cohort

Supplementary Table S2. Level of consciousness in the test cohort

Supplementary Table S3. Vital signs and symptoms in the test cohort

Supplementary Table S4. Comparison with other machine learning algorithms.

Supplementary Table S5. Prehospital stroke prediction for intervention using SPSS.

Figure S1. Study flowchart

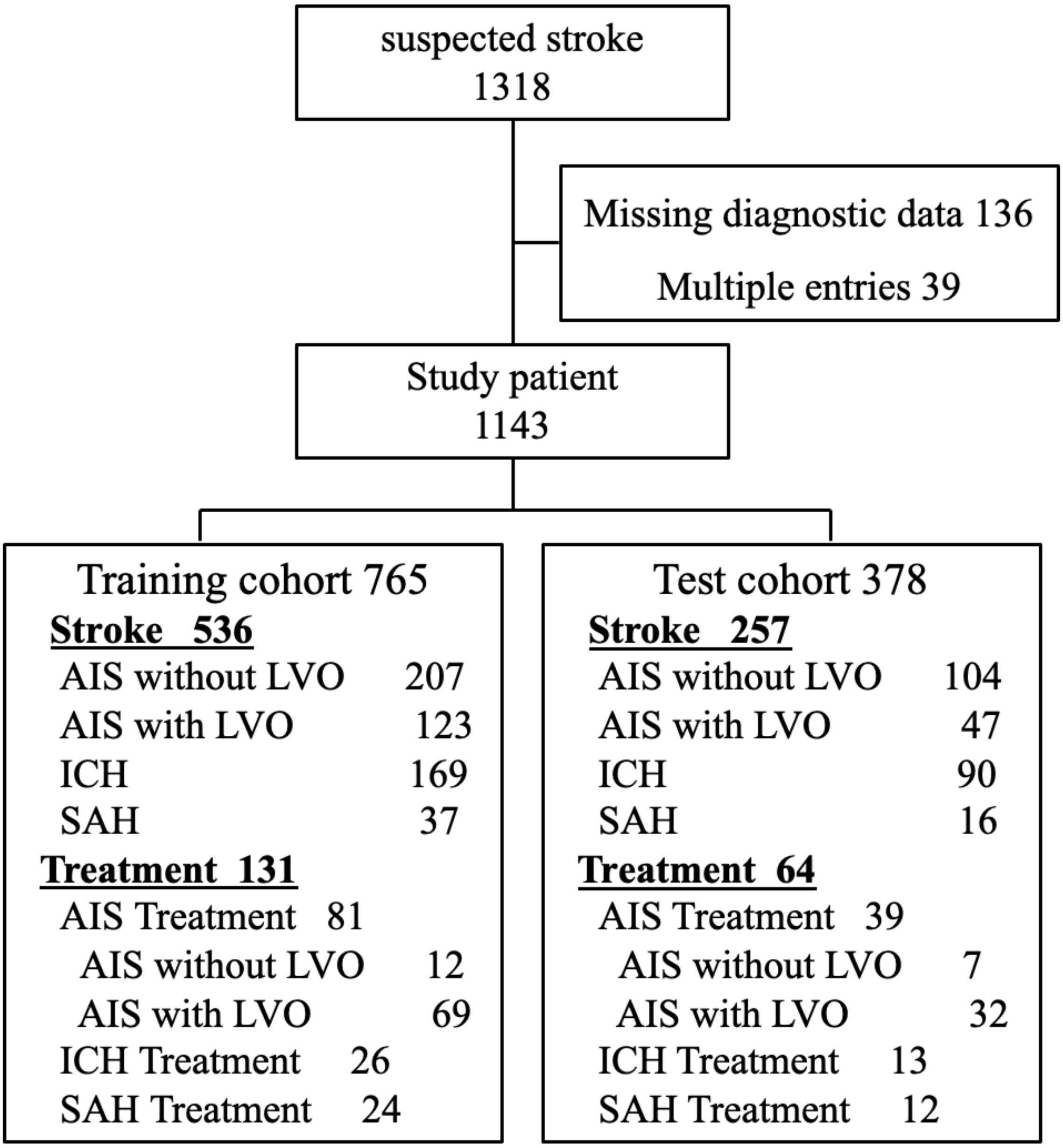

AIS; acute ischemic stroke, LVO; large vessel occlusion, ICH; intracranial hemorrhage, SAH; subarachnoid hemorrahage

Table S1. Baseline characteristics in the test cohort

	Treatment (N=64)	No treatment (N=314)	p value
Age, years	73.0 (62.0-81.5)	73.0 (61.0-82.0)	0.945
Male sex, n(%)	37 (57.8%)	197 (62.7%)	0.550
**Past medical history**			
Intracranial haemorrhage, n(%)	2 (3.4%)	18 (6.1%)	0.610
Anticoagulant/Antiplatelet therapy, n(%)	3 (5.9%)	34 (12.3%)	0.275
ADL indipendent, n(%)	50 (83.3%)	273 (89.8%)	0.221
Atrial fibrillation, n(%)	7 (12.1%)	12 (4.1%)	0.032
Hypertension, n(%)	30 (50.0%)	150 (49.8%)	1.000
Diabetes mellitus, n(%)	7 (11.7%)	41 (13.8%)	0.821
Cerebral infarction, n(%)	9 (15.0%)	58 (19.5%)	0.523
**Time course and onset timing**			
Time from onset to emergency call	27.0 (9.0-67.0)	52.0 (11.0-276.8)	0.030
Onset timing Monday	10 (15.6%)	45 (14.3%)	0.942
Onset timing Tuesday	12 (18.8%)	50 (15.9%)	0.710
Onset timing Wednesday	11 (17.2%)	34 (10.8%)	0.222
Onset timing Thursday	5 (7.8%)	38 (12.1%)	0.442
Onset timing Friday	8 (12.5%)	48 (15.3%)	0.705
Onset timing Saturday	10 (15.6%)	40 (12.7%)	0.675
Onset timing Sunday	8 (12.5%)	59 (18.8%)	0.307

Data are presented as median and interquartile range for continuous variables.
P-values were calculated using Pearson's chi-square test or the Mann–Whitney U test.

Table S2. Level of consciousness in the test cohort

	Treatment (N=64)	No treatment (N=314)	p value
**Japan Coma Scale**			
JCS 0, n(%)	20 (31.2%)	161 (51.3%)	0.005
JCS I-1, n(%)	11 (17.2%)	34 (10.8%)	0.222
JCS I-2, n(%)	3 (4.7%)	27 (8.6%)	0.423
JCS I-3, n(%)	16 (25.0%)	46 (14.6%)	0.064
JCS II-10, n(%)	7 (10.9%)	8 (2.5%)	0.005
JCS II-20, n(%)	0 (0.0%)	1 (0.3%)	1.000
JCS II-30, n(%)	1 (1.6%)	3 (1.0%)	1.000
JCS III-100, n(%)	4 (6.2%)	10 (3.2%)	0.412
JCS III-200, n(%)	1 (1.6%)	12 (3.8%)	0.598
JCS III-300, n(%)	1 (1.6%)	9 (2.9%)	0.869
**Glasgow Coma Scale**			
GCS (E)=4, n(%)	44 (68.8%)	259 (82.5%)	0.019
GCS (E)=3, n(%)	13 (20.3%)	19 (6.1%)	<0.001
GCS (E)=2, n(%)	1 (1.6%)	9 (2.9%)	0.869
GCS (E)=1, n(%)	6 (9.4%)	26 (8.3%)	0.968
GCS (V)=5, n(%)	23 (35.9%)	169 (53.8%)	0.013
GCS (V)=4, n(%)	14 (21.9%)	53 (16.9%)	0.439
GCS (V)=3, n(%)	8 (12.5%)	21 (6.7%)	0.182
GCS (V)=2, n(%)	6 (9.4%)	22 (7.0%)	0.691
GCS (V)=1, n(%)	13 (20.3%)	44 (14.0%)	0.275
GCS (M)=6, n(%)	41 (64.1%)	250 (79.6%)	0.011
GCS (M)=5, n(%)	12 (18.8%)	23 (7.3%)	0.008
GCS (M)=4, n(%)	5 (7.8%)	12 (3.8%)	0.283
GCS (M)=3, n(%)	1 (1.6%)	3 (1.0%)	1.000
GCS (M)=2, n(%)	2 (3.1%)	5 (1.6%)	0.749
GCS (M)=1, n(%)	3 (4.7%)	15 (4.8%)	1.000

JCS (Japan coma scale), GCS (Glasgow coma scale).
Data are presented as median and interquartile range for continuous variables.

P-values were calculated using Pearson's chi-square test or the Mann–Whitney U test.

Table S3. Vital signs and symptoms in the test cohort

	Treatment (N=64)	No treatment (N=314)	p value
**Vital signs**			
Heart rate	78.0 (65.0-90.0)	84.0 (74.0-96.0)	0.002
Arrhythmia	21 (38.9%)	46 (17.2%)	<0.001
Systolic blood pressure	164.0 (147.5-191.0)	172.0 (150.0-195.0)	0.441
Diastolic blood pressure	96.0 (79.2-104.0)	96.0 (82.0-114.0)	0.409
Body temperature	36.4 (36.0-36.6)	36.5 (36.2-36.8)	0.063
Oxygen Saturation	97.5 (96.0-99.0)	98.0 (96.0-98.0)	0.899
**Symptoms**			
Vomiting, n(%)	14 (22.6%)	38 (12.3%)	0.055
Dizziness, n(%)	8 (17.0%)	27 (10.5%)	0.299
Numbness, n(%)	6 (13.6%)	53 (22.5%)	0.264
Convulsion, n(%)	0 (0.0%)	17 (5.6%)	0.113
Upper limb paralysis, n(%)	43 (76.8%)	159 (55.0%)	0.004
Hemiparalysis, n(%)	34 (59.6%)	117 (39.9%)	0.009
Conjugate deviation, n(%)	16 (30.2%)	37 (13.3%)	0.004
Facial palsy, n(%)	22 (51.2%)	72 (32.7%)	0.033
Aphasia, n(%)	15 (34.1%)	51 (21.0%)	0.088
Dysarthria, n(%)	20 (55.6%)	105 (49.1%)	0.589
Unilateral spatial neglect, n(%)	4 (21.1%)	8 (6.1%)	0.071
Sudden headache or unconsciousness, n(%)	44 (68.8%)	115 (36.6%)	<0.001
Sudden headache, n(%)	17 (33.3%)	13 (6.7%)	<0.001

Data are presented as median and interquartile range for continuous variables.
P-values were calculated using Pearson's chi-square test or the Mann–Whitney U test.

Table S4. Comparison with other machine learning algorithms.

	AUROC	Accuracy	Sensitivity	Specificity	F1-score
Training cohort					
Logistic Regression	0.735 (0.691-0.781)	0.638 (0.607-0.671)	0.763 (0.689-0.835)	0.611 (0.576-0.648)	0.419 (0.365-0.476)
Random Forest	0.882 (0.853-0.913)	0.766 (0.735-0.795)	0.862 (0.796-0.924)	0.746 (0.711-0.779)	0.558 (0.499-0.619)
SVM(RBF)	0.727 (0.682-0.770)	0.647 (0.614-0.682)	0.710 (0.633-0.790)	0.634 (0.597-0.673)	0.408 (0.351-0.468)
Test cohort					
Logistic Regression	0.743 (0.682-0.801)	0.613 (0.561-0.667)	0.750 (0.642-0.847)	0.586 (0.530-0.648)	0.397 (0.317-0.474)
Random Forest	0.765 (0.703-0.824)	0.722 (0.677-0.767)	0.719 (0.606-0.825)	0.722 (0.673-0.772)	0.467 (0.382-0.544)
SVM(RBF)	0.638 (0.569-0.706)	0.608 (0.558-0.659)	0.531 (0.403-0.653)	0.624 (0.571-0.680)	0.310 (0.234-0.393)

AUROC; area under the receiver operating characteristic curve.

Table S5. Prehospital stroke prediction for intervention using SPSS.

AUROC	Accuracy	Sensitivity	Specificity	F1-score
0.587	0.481	0.747	0.427	0.329

Shonan Prehospital Scale; SPSS, AUROC; area under the receiver operating characteristic curve.
